# Exploring youth consumer behavior in the context of mobile short video advertising using an extended stimulus–organization–response model

**DOI:** 10.3389/fpsyg.2022.933542

**Published:** 2022-09-02

**Authors:** Kun Tian, Wenxia Xuan, Lijie Hao, Wenjing Wei, Dongping Li, Lu Zhu

**Affiliations:** ^1^College of Digital Arts, Communication University of Shanxi, Taiyuan, China; ^2^Graduate School of Business and Advanced Technology Management, Assumption University Thailand, Bangkok, Thailand; ^3^Shanxi Bethune Hospital, Tongji Shanxi Hospital, Third Hospital of Shanxi Medical University, Shanxi Academy of Medical Sciences, Taiyuan, China; ^4^China Australia Business College in Shanxi, Jinzhong, China

**Keywords:** mobile short video advertising, stimulus–organism–response theory, youth consumer, psychological satisfaction, consumer behavior

## Abstract

Under the hit of the epidemic, an increasing number of young people exchange and purchase goods by watching and resorting to mobile short video advertisements. Therefore, it is of great significance to explore the influence mechanism of mobile short video advertising on the consumption behavior of young people. This study develops a theoretical framework including fashion, socialization, entertainment, personalization, brand, psychological needs, satisfaction, and consumption behavior using a stimulus–organism–response (SOR) theory. The data from 332 young people using mobile short video advertising revealed that psychological needs exerted significant impacts on satisfaction, mediating the advertisements, and consumer satisfaction. The attributes of advertising, including fashion, socialization, entertainment, personalization, and branding, significantly promote young people’s psychological needs and satisfaction. In addition, satisfaction affects consumer behavior, and in the same manner, fashion and brand attribute directly impact consumer satisfaction.

## Introduction

The mobile short video is a new communication media using smartphones as the medium. It is characterized by timeliness, interaction, socialization, fragmented time consumption, independent fashion, and creative expression (Alamäki et al., 2019; Liu et al., 2019a; Choi et al., 2021). In recent years, with the prevalence of 5G technology, Mobile short videos have become an important part of people’s lives. Compared with other media, such as traditional TV, newspaper, and so on, advertisements on the platform of short video (fast dissemination, strong coverage, and high degree of interaction) has been recognized by many businesses. By using Internet technology to analyze and predict mobile short video advertisements, it cannot only allow users to browse their favorite product advertisements and improve user experience but also effectively improve the content and form of advertisements based on user needs, and promote consumers to generate purchase intentions and behavior (Wang M. L. et al., 2020). As a consequence, short videos have become a leading carrier of advertising information transmission and mainstream for merchants to implant advertisements for product promotion, it is also an important complement to existing advertising formats. Young, energetic, and fashionable young people are among the main consumer groups and generally, search for and select what they need by using short video advertisements in apps (Mu, 2020). At present, the number of students in colleges and universities reaches 40.2 million in China (The 2019 National Statistical Bulletin or Education Development, 2020), among whom 87.65% prefer shopping online (The 46th China Internet Statistics Report, 2021), showing great potential in commodity consumption.

As mobile video advertising occupies an increasing proportion of commercial advertising (Krämer et al., 2019), the correlation between mobile short video advertising and consumer buying behavior has been investigated from different dimensions. In short video advertisements, the characteristics of Internet celebrities and their opinions can significantly improve the intentions and attitudes of consumers (Liu et al., 2019b). Meng et al. (2020) found that the information source characteristics of Internet celebrities have an impact on arousing the search and purchase behaviors of consumers. Wang B. et al. (2020) believed that the design and presentation of short video advertisements should focus on user experience to meet psychological needs and thus improve the consumption willingness of consumers. For example, The bullet-screen advertising and two-dimensional design of Bilibili (China) cater to the psychological needs of young consumers, thus improving the click-through rate of advertisements. In recent years, new exploration has been made on short video advertisements and consumer interaction, revealing the influence of effective interaction between consumers and short video advertisements on purchase intention (Wang et al., 2018; Krämer et al., 2019). In previous studies, scholars have explored the relationship between short video advertising and consumer behavior and found influencing factors of different dimensions. In this study, the research objects mainly focus on the content attributes of mobile short video advertisements and consumer groups of young people. Therefore, this study is based on the theoretical model of stimulus–organism–response (SOR), searching large amounts of literature reviews of related studies. This study aimed at exploring the impact of new potential attributes of mobile short videos on the psychological satisfaction of young consumers. Besides, it also investigated how it influences consumption willingness, leading to the construction of a new theoretical model of online consumption suitable to the young consumers in the era of mobile Internet. This not only supplements and enriches the existing advertising theoretical models, but also provides a reference for the innovation and sustainable development of mobile short video advertising in the Internet era, and injects new vitality into the sustainable development of the consumer economy in the post-pandemic era.

Compared with traditional video advertising, short video advertising contains more information but takes less time. By accurate implantation into different fragmented periods of consumers, it can build product brand image through long duration and repetitive play to stimulate the desire of consumers to buy. Mobile short video advertising is a highly comprehensive form of advertising performance with social and entertainment attributes (The 2019 National Statistical Bulletin or Education Development, 2020; Zeng et al., 2009; Tsai and Men, 2013) and the characteristics of individuation, branding, and fashion (Simmonds et al., 2020; Teona et al., 2020). Young students are curious about novelties and satisfy their psychological needs by exploring interesting information and things (Portnoy et al., 2014). All these factors have an impact on the satisfaction and consumption of young students to a certain extent (Luo and Homburg, 2007). This study proposes that the seven attributes, namely, socialization, entertainment, personalization, fashion, brand, psychological needs, and satisfaction attributes are the key features of mobile short video advertising. On this basis, this study applied SOR theory (Mehrabian and Russell, 1974) to study the consumption of college students and discussed the influence of mobile short video advertisements on their purchase intention and corresponding mechanism according to the seven attributes.

## Literature review and hypotheses development

### Theoretical background

The theoretical framework of the present study is developed based on the adaptation of the SOR model (Mehrabian and Russell, 1974) and the consumer behavior literature. Moreover, we adopted the Uses and Gratifications Theory (Severin and Tankard, 1997) and Model of Information System Success (DeLone and McLean, 1992) to rationalize and explain the cause and effect relationship among variables. The classical SOR model has been applied in various retail settings to explain consumer decision making (Richard et al., 2009). In the information age, online retailing has emerged as the most rapidly growing retail form (Mulpuru et al., 2011) and has been focused on various aspects using the SOR framework. For example, Richard and Chandra (2005) investigated the network environment, website navigational features, user characteristics, internal states, consumer responses, and outcomes in online communication. Mummalaneni (2005) emphasized the influence on relationships among website characteristics, emotional responses, and purchasing behaviors of consumers. Gong et al. (2022) discovered that the sense of risk to the Internet from consumers and their trust in the platform can have a significant influence on their trust in the brand, which can positively influence consumer willingness. Moreover, the extended SOR model was utilized to predict consumer behavior by incorporating additional variables such as cognition and perceived service quality (Kim and Moon, 2009; Vieira, 2013; Gong et al., 2022). More importantly, with the upgraded SOR theoretical model, multiple stimuli have been studied extensively, including social support (Zhang et al., 2014), flow (Animesh et al., 2011; Zhang et al., 2014), sensation (Vieira, 2013; Kim and Johnson, 2016), and interaction (Zhao and Lu, 2012). This allows the model to be used in different scenarios and further refined. According to the SOR model, all behavioral outcomes contain the results obtained by the interaction with the external environment and the internal psychological manifestations of humans after obtaining results (Illeris, 2003). The Uses and Gratifications Theory and Model of Information System Success include information quality, personal influence, social organization impact, user cognition, and consumer satisfaction, which can significantly enrich the framework of SOR. With the widespread usage of media, this application is defined as social behavior. The values behind its usage can be divided into utilitarian value and enjoyment value (Stephenson, 1967; Renckstorf et al., 2004). Especially in the Internet era, with the change in consumption environment (from the physical environment to complex social interactions) and consumer behavior (from individual behavior to group interactions), the relationship between the above two values can be extremely complicated. However, these values can be reflected in their psychological needs, when they use mobile short videos. Satisfaction of this psychological need can improve user satisfaction, which produces consumption behavior as a result. Consequently, we argue that users perceive multifactorial stimuli when using mobile short video advertisements. Such stimuli can influence their psychological needs and satisfaction to generate consumption behaviors. Based on this, we believe that the SOR model can provide a theoretical basis for this study.

Psychological needs result from continuous interaction between users and external stimuli (Illeris, 2003). The psychological satisfaction of users is a behavioral indicator of their satisfaction and loyalty to information systems (Cheung et al., 2015; Khan, 2017). In this study, the Uses and Gratifications Theory was used to support the framework of SOR. When the user expectation and psychological needs are satisfied, In the process of consumption, when consumers’ expectation and psychological needs are met, consumers will be stimulated to consume as a consequencce. Without them, the consumption will expire (Rauniar et al., 2013). Mobile short video advertising could present information containing many social, personal, and other related factors, influencing the psychological expectations, satisfaction, and consumption behaviors of consumers (Kim et al., 2013). Socialization, entertainment, and psychological demands are the main drivers of consumer behaviors (Park et al., 2009; Revels et al., 2010). Moreover, mobile short video advertisements are also characterized by personalization and fashion to attract consumers to generate potential interest and consumption behavior. Some analyses have been conducted on the attributes of mobile short video advertisements, but limitations were unavoidable due to the lack of social, personal, and other relevant latitudinal factors. This study developed and tested an extended SOR model to predict the psychological needs and consumption behaviors of young users of mobile short video advertisements (Cheng et al., 2013).

### Hypothesis development

Through sorting out relevant literature on mobile short video advertising, this study summarizes the attributes of the advertising of different dimensions. On this basis, the influence of mobile short video advertising on the consumption intention of young college students and its corresponding mechanism are explored.

#### Socialization

Socialization means that consumers or users purchase products and establish connections with each other (Zhang et al., 2014) which serves as an attribute of mobile short video advertising. Social media platforms such as Tik Tok (China), Xiaohongshu (China), and Weibo present advertising content with fashion elements. Moreover, it provides a platform for businesses and consumers to display, share, and discuss fashion content and topics (Ge et al., 2021). In this process, college students with consistent hobbies will build “circles” to share their preferences and relevant fashion information to meet psychological needs and achieve consumption intention. This will also bring young consumers a sense of physical and mental pleasure and satisfaction. Based on the above contents, this study proposes the following hypothesis:

**Hypothesis 1 (H1)**. *The socialization of mobile short video advertisements has a positive impact on the psychological needs of young consumers.*

#### Entertainment

The second attribute of mobile short video advertisements is entertainment. Entertainment refers to the enjoyment and pleasant psychological emotions generated in consumers or users during and after using products (Huang, 2017; Wang and Genç, 2019). Young college students watching short video advertisements will choose the information content according to their interests and preferences. The continuous pleasantness of psychological mood derived from desired information content will influence their consumption satisfaction. Similarly, Chen et al. (2014) and Gao and Zang (2016) found that the entertainment content of information products could attract the intention of consumers to use and participate and significantly influence their continuous participation and consumption motivation. Therefore, the highly entertaining fashion advertising content will bring about a sense of pleasure to college students and young people during consumption and increase their psychological satisfaction and consumption intention. Therefore, the following hypothesis is proposed:

**Hypothesis 2 (H2)**. *The entertainment of mobile short video advertisements has a positive influence on the psychological needs of young consumers.*

#### Personalization

Personalization is the third attribute of mobile short video advertisements. It refers to the degree of conformity between information content and personal preferences (Chen et al., 2014; Gao and Zang, 2016). With the advancement of information technology, advertisers rely on big data information to provide advertising content for targeted users to meet their needs (Portnoy et al., 2014). In this process, consumers will take the initiative to invest more time and energy to screen the information and purchase needed goods, thus producing consumption behavior. Compared with social consumption groups, college students present more personalized consumption. They will pay more attention to the commodity itself and personalized service proactively. Through purchasing and using personalized goods, they display their personality and generate circle worship to satisfy their psychological needs. Therefore, hypothesis 3 is proposed:

**Hypothesis 3 (H3)**. *The personalization of mobile short video advertisements exerts a positive influence on the psychological needs of young consumers.*

#### Fashion

The fourth attribute of mobile short video advertisements is fashion. Fashion refers to something in an established state in a certain period with admiration from a considerable number of consumers (Cai et al., 2019). Generally, consumers are satisfied by acquiring goods admired and recognized by social groups. Young people are fashionable and energetic and have unique demands for fashion content. Therefore, the young consumer groups pay more attention to fashion characteristics and content diversification of goods online. Moreover, to cater to the psychological demands of college students and young people for fashion elements and stimulate consumer behavior, online merchants elaborately create short video advertisements from the commodity itself, copywriting, design, packaging, and other aspects. On this basis, the following hypotheses are proposed:

**Hypothesis 4 (H4)**. *The fashion attribute of mobile short video advertisement has a positive influence on the psychological needs of young consumers.*

**Hypothesis 5 (H5).** *The fashion attribute of mobile short video advertisements has a positive impact on the satisfaction of young consumers.*

#### Branding

The fifth attribute of mobile short video advertisements is branding. The product brand is an important factor affecting consumer behavior and is influenced by the price and quality of products (Okazaki, 2009; Li and Zhang, 2020). The quality, price, and trend of goods are the main factors affecting the consumption of young people (Lin et al., 2015; Lei, 2017). Restricted by economic conditions, most young people tend to buy cheap goods to meet their psychological needs. However, they are vulnerable to the potential influence of the surrounding environment and other factors, resulting in the psychology of brand pursuit and consumption of goods with brand attributes. Based on this, the following hypotheses are proposed:

**Hypothesis 6 (H6).** *Branding of mobile short video advertisements has a positive influence on the psychological needs of young consumers.*

**Hypothesis 7 (H7).** *Branding of mobile short video advertisements has a positive impact on the satisfaction of young consumers.*

#### Psychological needs

The sixth attribute of mobile short video advertisements is psychological needs. Based on the consumption process in modern society, consumer behavior is a kind of psychological need of consumers (Liu and Wan, 2018; Li and Atkinson, 2020). Consumers with potential psychological demands for consumption choose required goods through short videos, during which they interact with short video content, and their psychology and emotion fluctuate with the changes in content, these contents affect consumer satisfaction (Oliver, 1993). Consumption behavior occurs when consumers interact with objects attractive for them to meet their motivations and needs (Xiong and He, 2017). In this process, the change of psychological needs can have an impact on consumer satisfaction with the whole short video advertising. Therefore, psychological needs have a positive effect on the satisfaction of college students. Similarly, the psychological needs and satisfaction of young people, a special consumer group, are influenced by different factors simultaneously (Houghton et al., 2020). However, psychological needs can mediate the occurrence of other behavioral attributes affecting the satisfaction of young people. On this basis, the following hypotheses are proposed:

**Hypothesis 8 (H8)**. *Psychological needs have a positive impact on the satisfaction of young people.*

**Hypothesis 9 (H9)**. *Psychological needs play an intermediary role in the relationship between mobile short video advertising factors and the satisfaction of young people.*

**Hypothesis 9_*a*_ (H9_*a*_)**. *Psychological needs play a mediating role between fashion and the satisfaction of young people.*

**Hypothesis 9_*b*_ (H9_*b*_)**. *Psychological needs mediate the relationship between socialization and the satisfaction of young people.*

**Hypothesis 9_*c*_ (H9_*c*_)**. *Psychological needs mediate the relationship between entertainment and the satisfaction of young people.*

**Hypothesis 9_*d*_ (H9_*d*_)**. *Psychological needs mediate the relationship between personalization and the satisfaction of young people.*

**Hypothesis 9_*e*_ (H9_*e*_)**. *Psychological needs mediate the relationship between brand and the satisfaction of young people.*

#### Satisfaction

In the process of understanding human intention and behavior, one of the fundamental elements is to comprehend human needs (Deci and Ryan, 2000). Use and Gratification Theory defines how and why individuals turn to some media for certain needs. Maslow (1958) defined human needs as a driving force. When human needs have been satisfied to a certain level, the corresponding behavior can be conducted. However, the need for satisfaction is not limited physically, but spiritually. Both the physical needs and spiritual needs can be overlaid and accumulated (Kenrick et al., 2010). There is existing research indicating that need satisfaction can improve consumer satisfaction (Luo and Homburg, 2007), which can promote the consumption behavior of consumers (Hansemark and Albinsson, 2004). Therefore, it is believed that with the satisfaction of young consumers with the usage of mobile short videos, their consumption behavior can be produced consequently. Based on this, the following hypotheses are proposed:

**Hypothesis 10 (H10)**. *Satisfaction has a positive impact on the consumption behavior of young people.*

### Construction of the conceptual model

**FIGURE 1:**
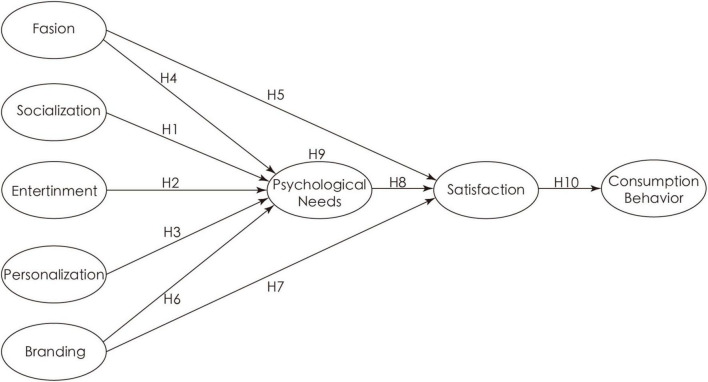
Model construction.

Based on the SOR model, this article intends to explore the influence of different types of short video advertising factors on the consumption intention of young people, as shown in Figure 1.

## Methodology

### Data collection

In this article, questionnaires are designed on the platform of Wenjuanxing (China), and data resources are obtained by issuing questionnaires to young college students using mobile short videos. Each respondent is randomly allocated a red envelope ranging from 5 to 10 RMB. A total of 378 questionnaires were collected in this study, among which 46 received unclear answers, incomplete filling, and less than 2 min were deleted. The final number of valid questionnaires was 332. The sample description of the questionnaire is as follows: 124 male users, accounting for 37.63%; 208 female users, covering 62.6%; 94.0% of users are aged 18–26 years; and 98.9% of users obtained a college degree or above. The average time of daily Internet use was 6.55 h, of which 3.04 h were spent watching short videos on apps. A total of 94.5% of users spend less than 3,000 RMB on fashion products every month, of which 97.1% pay attention to fashion products less than 1,000 RMB. From the demographic characteristics of the data sample, this sample is well represented.

### Measurement of variables

All scales used in this study referred to scales similar to those studied at home and abroad and were tested with good reliability and validity. For the measurement of socialization and personalization, the study of Zhang et al. (2014) was used for reference, with three and four selected items, respectively. The measurement of fashion adopted the research by Sundar et al. (2014), which included four items. In terms of entertainment, Lin and Hsieh (2011) utilized three selected items for measurement. In response to psychological needs and consumer behaviors, the scales of Fang et al. (2017) were used for reference, with three and four selected items, respectively, for measurement. As for the measurement of user satisfaction, Bhattacherjee et al. (2008) questionnaire was referred to and adopted, using three choices to measure. The evaluation of the brand attribute used the study of Kim and Parker (1999) for reference with four-choice questions for measurement. The dimensions of measurement items were set according to five-item Likert scales, which were divided into five levels, namely, strongly disagree, disagree, undecided, agree, and strongly agree, with the assigned values of 1, 2, 3, 4, and 5, respectively. The items in the questionnaire are shown in Table 1.

**TABLE 1:** Variable scale and items of mobile short video advertisements.

Variables	Factors	Items	References
Socialization	S1	Short video ads allow me to build good social relationships with other fashionistas	Zhang et al., 2014
	S2	Short video ads make me feel like I’m part of the fashion community	
	S3	Short video ads allow me to form close friendships with fashionistas	
	S4	Short video ads leave me a good impression of the fashion people around me	
Fashion	F1	Short video advertising content can make viewers look more stylish	Sundar et al., 2014
	F2	If I watch short video ads, it makes me more stylish	
	F3	Short video ads help people who watch them show fashion	
	F4	People who watch short video ads have unique personalities	
	F5	People who watch ads that share short videos are considered fashion setters	
Entertainment	E1	The content of short video ads is presented in an interesting way	Lin and Hsieh, 2011
	E2	Watching short video ads makes me feel good	
	E3	Short video ads have some interesting add-ons	
Personalization	P1	Short video ads know what I want	Zhang et al., 2014
	P2	Short video ads understand my specific fashion needs	
	P3	Short video ads store my favorite fashion content and offer additional services based on my preferences	
	P4	Short video ads do a great job of guessing what fashion content I want and making suggestions	
Branding	B1	When buying goods, I will consider the goods with high cost performance	Kim and Parker, 1999
	B2	When buying goods, I will consider the value of goods	
	B3	Fashion items are reasonably priced when viewing short video ads	
	B4	When buying goods, buy fashionable goods at reasonable prices	
Psychological needs	PN1	Short video ads can let me get more fashion information	Fang et al., 2017
	PN2	Short video ads can let me know more about fashion activities	
	PN3	Short video ads allow me to get more useful information about fashion goods and services	
Satisfaction	S1	Watching short fashion video ads makes me feel very happy.	Bhattacherjee et al., 2008
	S2	Watching fashion short video ads make my needs satisfied.	
	S3	Watching short video ads has been very satisfying for me overall.	
Consumption behavior	CB1	I want to watch and use short video advertising content	Fang et al., 2017
	CB2	I plan to watch short video ads to buy fashion products in the future	
	CB3	I will recommend other people watch short video advertising messages	
	CB4	If my friends and relatives are looking for consumer advice, I will recommend watching short video ads	

Because data were collected in China, translation and back-translation were resorted to ensure the translation quality. First, we consulted two linguistic professors to comprehend the significance and readability of each item. The English questionnaire was then translated into Chinese with their help. Second, the Chinese questionnaire was translated into English by two students with master’s degrees. Third, we compared the translated items with the original English version. To ensure the consistency of the two English versions, we polished the translation and eliminated all inconsistencies.

## Results

### Reliability and validity test

The Cronbach’s alpha average values of all scales in this study were above 0.9, indicating the high reliability of the scale. Confirmatory factor analysis was used to test the scale for discriminant validity and convergent validity, and items with a factor load lower than 0.5 were deleted. Finally, the standardized factor load of each question was greater than 0.5, the square-root extract average variance extracted (AVE) was greater than 0.5 (Fornell and Larcker, 1981), and the composite reliability (CR) was greater than 0.7, indicating a good convergence validity of the variable. The square-root AVE values of all variables were greater than the relational values of this variable and other variables, indicating the satisfying discriminant validity of the scale, as shown in Tables 2, 3.

**TABLE 2:** Model reliability and validity analysis.

Variable	Item	Standardized factor load	AVE	CR	Cronbach’s alpha
Socialization	S1	0.949	0.866	0.963	0.962
	S2	0.935			
	S3	0.928			
	S4	0.912			
Fashion	F1	0.910	0.798	0.922	0.919
	F2	0.927			
	F3	0.842			
Entertainment	E1	0.933	0.858	0.947	0.948
	E2	0.925			
	E3	0.921			
Personalization	P1	0.875	0.795	0.939	0.939
	P2	0.916			
	P3	0.915			
	P4	0.861			
Branding	B1	0.914	0.777	0.933	0.933
	B2	0.918			
	B3	0.844			
	B4	0.848			
Psychological needs	PN1	0.897	0.826	0.934	0.934
	PN2	0.932			
	PN3	0.898			
Satisfaction	S1	0.791	0.635	0.838	0.819
	S2	0.884			
	S3	0.707			
Consumption behavior	CB1	0.836	0.719	0.911	0.910
	CB2	0.850			
	CB3	0.875			
	CB4	0.831			

**TABLE 3:** Square AVE values and correlation coefficients of each variable.

	B	P	E	S	F	PN	S	CB
B	0.881							
P	0.700	0.892						
E	0.718	0.655	0.926					
S	0.669	0.625	0.673	0.931				
F	0.733	0.715	0.678	0.638	0.888			
PN	0.837	0.759	0.819	0.765	0.766	0.909		
S	0.694	0.594	0.605	0.566	0.685	0.712	0.797	
CB	0.368	0.460	0.212	0.456	0.551	0.573	0.805	0.954

The diagonal is the square root of AVE.

### Hypothesis testing

**FIGURE 2:**
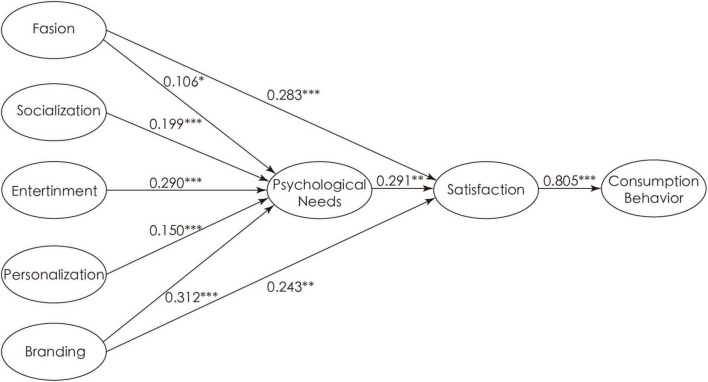
Hypothesis testing results. ^***^p-value < 0.001, ^**^p-value < 0.01, *p-value < 0.05.

AMOS 24.0 was used for structural equation model analysis, with model fitting χ^2^*/df* of 2.355 (less than 3), *RMSEA* of 0.064 (less than 0.80), *GFI* of 0.857 (close to 0.90), *CFI* of 0.955, and *NFI* of 0.925. Moreover, other indicators are greater than 0.90. This indicated the good fitting degree and acceptability of the model (Henseler et al., 2015). The results of the hypothesis are shown in Table 4. In the hypothesis test of the influence of socialization on psychological needs, the positive influence of socialization (β = 0.199, *P* < 0.001) was significant, supporting hypothesis 1. In the hypothesis test of the influence of entertainment and personalization on psychological needs, entertainment (β = 0.290, *p* < 0.001) and personality attributes (β = 0.150, *p* < 0.001) had a positive and significant impact, testifying hypotheses 2 and 3. In the hypothesis test of the influence of fashion on psychological needs and satisfaction, fashion (β = 0.106, *p* = 0.035 < 0.05; β = 0.283, *p* < 0.001) had a significant and positive effect, verifying hypotheses 4 and 5. In the hypothesis test of the influence of branding on psychological needs and satisfaction, brand (β = 0.312, *p* < 0.001; β = 0.243, *p* = 0.008 < 0.01) had a significant and positive effect, confirming hypotheses 6 and 7. Finally, psychological needs had a positive influence on satisfaction (β = 0.291, *p* = 0.003 < 0.01), and satisfaction had a positive influence on consumption behavior (β = 0.805, *p* < 0.001), confirming hypotheses 8 and 10. Taken together, all hypotheses are significantly valid, and the structural equation model is shown in Figure 2.

**TABLE 4:** Results of structural equation model.

Hypothesis	Structural equation path	Path analysis	*t*-value	*P*-value	Result
H_1_	S > > PN	0.199	4.702	0.001***	Support
H_2_	E > > PN	0.290	6.149	0.001***	Support
H_3_	P > > PN	0.150	3.228	0.001***	Support
H_4_	F > > PN	0.106	2.112	0.035*	Support
H_5_	F > > S	0.283	3.702	0.001***	Support
H_6_	B > > PN	0.312	5.950	0.001***	Support
H_7_	B > > S	0.243	2.632	0.008**	Support
H_8_	PN > > S	0.291	2.946	0.003**	Support
H_10_	S > > CB	0.805	13.208	0.001***	Support

***p-value < 0.001, **p-value < 0.01, *p-value < 0.05.

In this study, AMOS24.0 was used to analyze the mediating effect of psychological satisfaction on the relationship between the consumption attributes and consumption behaviors of college students. Bootstrap was set to 5,000 times to build confidence interval (Hayes, 2009). As shown in Table 5, the results showed that psychological needs had significant mediating effects on attributes of fashion, entertainment, personalization, branding, and consumer behavior (*p* < 0.05), supporting hypotheses 9a–e.

**TABLE 5:** Analysis of results of mediation effect.

Hypothesis	Mediating path	Coefficient	95% confidence interval	*P*-value	Result
H_9a_	F > > PN > S	0.296	0.164–0.434	0.014*	Support
H_9b_	S > > PN > > S	0.126	0.058–0.207	0.001***	Support
H_9c_	E > > PN > > S	0.064	0.009–0.131	0.036*	Support
H_9d_	P > > PN > > S	0.101	0.045–0.170	0.001***	Support
H_9e_	B > > PN > > S	0.281	0.146–0.418	0.001***	Support

***p-value < 0.001, **p-value < 0.01, *p-value < 0.05.

## Discussion and conclusion

Based on SOR theory, this study investigated the influence of the attributes of mobile short video advertising on the consumption behavior of young people through literature review and analysis. The results showed that the socialization, fashion, entertainment, personalization, and branding attributes of mobile short video advertising had positive impacts on the psychological needs of young people. Among them, fashion, branding, and psychological needs have a positive influence on the satisfaction of young people, the satisfaction directly affects consumer behavior.

The findings of this study are expected to make the following contributions. First, based on the SOR theoretical model, multiple paths of different attributes of mobile short video advertisements on psychological needs, satisfaction, and young people’s consumption behavior are constructed. Under the influence of different attributes, the relationship between these attributes and the psychological needs and consumption behavior of young people is analyzed. Besides, this study explored and discussed new mobile short video attributes and verified the previous research results (Stephenson, 1967; Renckstorf et al., 2004; Wang M. L. et al., 2020). Second, psychological needs are taken as the mediating variable of the influence of mobile short video advertisement attributes on the satisfaction and consumption behavior of young people, further enriching the framework of relations among attributes of mobile short video advertising, consumer psychological needs, and consumption behavior, providing a new perspective for research on the influence of mobile fashion short video advertisements on the consumption behavior of young people.

The consumption behavior of young people is affected by both external and internal factors. The interaction of the two types of factors will exert different effects on psychological needs (Severin and Tankard, 1997; Li and Atkinson, 2020). Psychological needs and satisfaction are the driving forces of consumption behaviors (Revels et al., 2010; Kim et al., 2013). Moreover, this study also confirms that different attributes of mobile fashion short video advertisements will significantly impact the psychological needs of young people. As a mediating mechanism in their satisfaction and consumption, psychological needs play a positive and significant role in the influence of socialization, fashion, entertainment, personalization, and branding attributes on satisfaction and consumption behavior. It can be seen that the consumption of young consumers is influenced by various factors and promoted by the satisfaction of psychological needs. During consumption, psychological satisfaction is affected by internal and external factors and acts on consumption behavior. However, we also found that fashion attributes and branding attributes in mobile short video advertisements directly affect consumer behavior, particularly young consumers. To sum it up, young consumers are more favorable to fashionable and brand merchandise.

### Practical implications

This article provides the following recommendations. First, the focus should be on advertising content and features should be highlighted from multiple angles. When merchants and business provide short video advertising for young people, they should give full consideration to their unique brand advantages and potential value to meet the demands of consumers for fashion, personalization, and branding based on positive and safe advertising content. In particular, the advertising content should cater to the psychological needs of young people, such as short video copywriting, video, personal preferences, pictures, sound effects, and other innovative elements, to meet their psychological needs and strengthen their loyalty constantly. In the meantime, with the application of IP effect, brand value, and its advantage, user needs can be gradually satisfied, which improves users’ interaction and increase customer stickiness.

Second, a “circle” culture is encouraged to be built with multi-dimensional interaction and communication. When presenting fashionable short video advertisements for young people, merchants can build a unique “circle” culture of fashion products through the entertainment and socialization behind advertisements and form a multi-dimensional exchange and interaction among young people, merchants, and fashion groups. As a result, merchants can directly and efficiently obtain the needs of young people for fashion content. Moreover, merchants and fashion groups can also transmit more fashion information to young consumers, promoting multi-dimensional (visual and auditory) information communication and interaction, satisfying the emotional belonging and existence of consumers when they use mobile short video advertising, which can generate resonance and induce feeling expression between consumers and business. Therefore, businesses must perfect the functions of advertising platforms, strengthening the interactions between virtual and reality, and satisfying the multi-needs of consumers.

Last but not least, this study advocates businesses to establish a new profit model, coconstructing the ecological chain model between business and consumers, accurately analyzing the consumption groups, lessening or deleting unfavorable advertisements, and designing more innovative and emotional advertisements, which can generate more consumption behaviors and promote the sustainable development of mobile short video advertising healthily.

Through this model, each young college student can play a leading role in the short video. Under the effect of their mutual influence, the psychological needs of young people can be satisfied, and thus their consumption behavior can be stimulated. Finally, a consumption pattern of “point to line, line to plane” will be formed to drive economic recovery and prosperity in the post-epidemic era from the consumption level.

## Research limitations

Our findings enrich the literature on mobile short video advertising factors, SOR models, and consumption intentions of young age groups. Nevertheless, there are some limitations. First, as the COVID-19 pandemic is under effective control, the shopping environment combining online and offline shopping will be a new development trend, and future studies should test the model in different scenarios, such as online perceptions and offline experiences, to complement each other. Second, the data used in this study are mostly collected from the young student group and do not involve numerous social groups of young people. Therefore, whether similar consumption behaviors can be generated under the influence of mobile short video advertising factors remain to be explored. Third, due to time and space constraints, the sample in this study may not accurately represent all groups of young people. For this reason, different regions should be included and compared in future research, such as the East and West, to expand the sample size and improve the representativeness of the study. By doing this, more insights will be provided in mobile short video advertising.

## Data availability statement

The original contributions presented in this study are included in the article/supplementary material, further inquiries can be directed to the corresponding author.

## Ethics statement

Ethical review and approval were not required for the study on human participants in accordance with the local legislation and institutional requirements. The patients/participants provided their written informed consent to participate in this study.

## Author contributions

KT wrote the draft of the manuscript. WX contributed to the samples of the research. LH played a major role in the technical support. DL and WW gave support to the data analysis. LZ gave guidance on the methodology. All authors contributed to the article and approved the submitted version.
